# Temperature Dependence of Interfacial Bonding and Configuration Transition in Graphene/Hexagonal Boron Nitride Containing Grain Boundaries and Functional Groups

**DOI:** 10.3390/ijms23031433

**Published:** 2022-01-27

**Authors:** Lei Fan, Wenjuan Yao

**Affiliations:** Shanghai Institute of Applied Mathematics and Mechanics, Shanghai University, Shanghai 200072, China; fanleigl@shu.edu.cn or

**Keywords:** hexagonal boron nitride matrix ceramic, functional groups and grain boundary, temperature, interfacial bonding, molecular dynamics

## Abstract

The quasi-three-dimensional effect induced by functional groups (FGo) and the in-plane stress and structural deformation induced by grain boundaries (GBs) may produce more novel physical effects. These physical effects are particularly significant in high-temperature environments and are different from the behavior in bulk materials, so its physical mechanism is worth exploring. Considering the external field (strain and temperature field), the internal field (FGo and GBs) and the effect of distance between FGs and GBs on the bonding energy, configuration transition, and stress distribution of graphene/h-BN with FGo and GBs (GrO-BN-GBs) in the interface region were studied by molecular dynamics (MD). The results show that the regions linked by hydroxyl + epoxy groups gradually change from honeycomb to diamond-like structures as a result of a hybridization transition from sp^2^ to sp^3^. The built-in distortion stress field generated by the coupling effect of temperature and tension loading induces the local geometric buckling of two-dimensional materials, according the von Mises stresses and deflection theory. In addition, the internal (FGo and GBs) and external field (strain and temperature field) have a negative chain reaction on the mechanical properties of GrO-BN-GBs, and the negative chain reaction increases gradually with the increase in the distance between FGo and GBs. These physical effects are particularly obvious in high-temperature environments, and the behavior of physical effects in two-dimensional materials is different from that in bulk materials, so its physical mechanism is worth exploring.

## 1. Introduction

Two-dimensional material is a kind of low-dimensional structure that has attracted much attention in recent years [1,2,3]. It consists of one or several layers of atoms arranged in two-dimensional space or quasi-two-dimensional space with a certain thickness [4,5,6]. From the point of view of material design, two-dimensional materials can be used as the basic unit for building fiber, film, and bulk materials [7,8,9]. Because all atoms in two-dimensional materials are exposed to the environment, the material design from bottom to top can be realized by using functional group and defect engineering [10]. In 2021, J. F. Rocha et al. [11] found graphene oxide microfibers with controlled and homogenous shapes and tunable diameters by using the three-dimensional hydrodynamic focusing concept on a microfluidic device. Their research indicates that the 3D hydrodynamic flow focusing microfluidic device may be extended to one- and two-dimensional materials to produce controlled hierarchical microfibers, by the controlled combination of different heterostructures to achieve multifunctional applications [11]. Therefore, graphene oxide and graphene heterostructures have expanded the functions and application fields of graphene [12,13], and laid a solid foundation for the application of new graphene materials in optoelectronic devices, biomedicine, and environmental and energy technology [14,15].

Graphene heterostructures have aroused great interest in the past decade due to their novel spatial configuration and unprecedented physical properties [16,17]. Most of these attractive properties are strongly dependent on the different spatial structures of heterostructures [18], usually including the vertical heterostructures that vertically stack multiple two-dimensional materials [19] and the in-plane heterostructures [20] that seamlessly stitch together two different atomic monolayers through covalent bonds [21]. Compared with vertically stacked heterostructures, in-plane heterostructures are rarely realized due to requirements of bottom-up synthesis. In addition, a certain number of topological defects inevitably occur in the process of material synthesis due to polymorphism and different bonding geometry in planar heterostructures [22,23]. These topological defects affect the physical and material properties of planar heterostructures [24,25].

In terms of mechanical properties, the existence of topological defects can cause stress accumulation in the material, which is more significant with the decrease in the structural geometric dimension [26]. Moreover, comparing the tensile strength of defect-free graphene sheets with that of existing engineering materials, the former is more than two orders of magnitude higher than the latter [27]. In addition, single-layer atomic structures are very prone to out-of-plane deformation, and internal defects inevitably appear at the interface of graphene/boron nitride heterostructures [28]. These unique mechanical behaviors brought by dimensional limits and new spatial structures are the key to solving the problems of reliability and durability in the process of applying graphene materials in engineering. 

Short-line stacking of topological defects or other special defects (functional groups, etc.) produces local stress fields, and its amplitude has a certain random distribution in actual material samples [29]. For graphene heterostructures with a large number of these independent and random defects, the strength is determined by the weakest part of the material.

It has been confirmed [30] that the interface region is one of the important factors affecting the physical properties of graphene/boron nitride (G-BN) configurations. However, it is not clear at present what influence the coexistence of FGo and GBs has on the physical properties of the interface region (key point) of G-BN configurations, and most importantly, what effect the coupling of temperature and strain rate has on the bonding energy and configuration transition of G-BN with FGo and GBs configurations. Considering temperature, strain rate, FGs type, FGs density, and the effect of distance between FGs and GBs on the bonding energy, the configuration transition and stress distribution of GrO-BN-GBs interface regions were studied by molecular dynamics. This could provide a reference and theoretical basis for the optimal design of two-dimensional materials with high mechanical properties.

## 2. Results and Discussion

### 2.1. Validation

To verify the simulation, Table 1 and Table 2 show the results of previous work compared with the three mechanical values (fracture stress, strain, and Young’s modulus) of graphene and boron nitride in this study. As shown in Table 1 and Table 2, the comparison shows that the calculation method and the selection of potential function were very reasonable, and the results obtained were very consistent with previous work [31]. The failure stress and the strain of the pristine graphene were 123 GPa and 0.204, respectively. In addition, the failure stress and the strain of the pristine h-BN were 125 GPa and 0.207, respectively. The Young’s modulus of the pristine h-BN (921 GPa) was in good agreement with the experimental result (881 GPa [32]) and the simulation result (930 GPa [28]).

### 2.2. Coupling Effect of GBs and FGs at High Temperature

**Figure 1:**
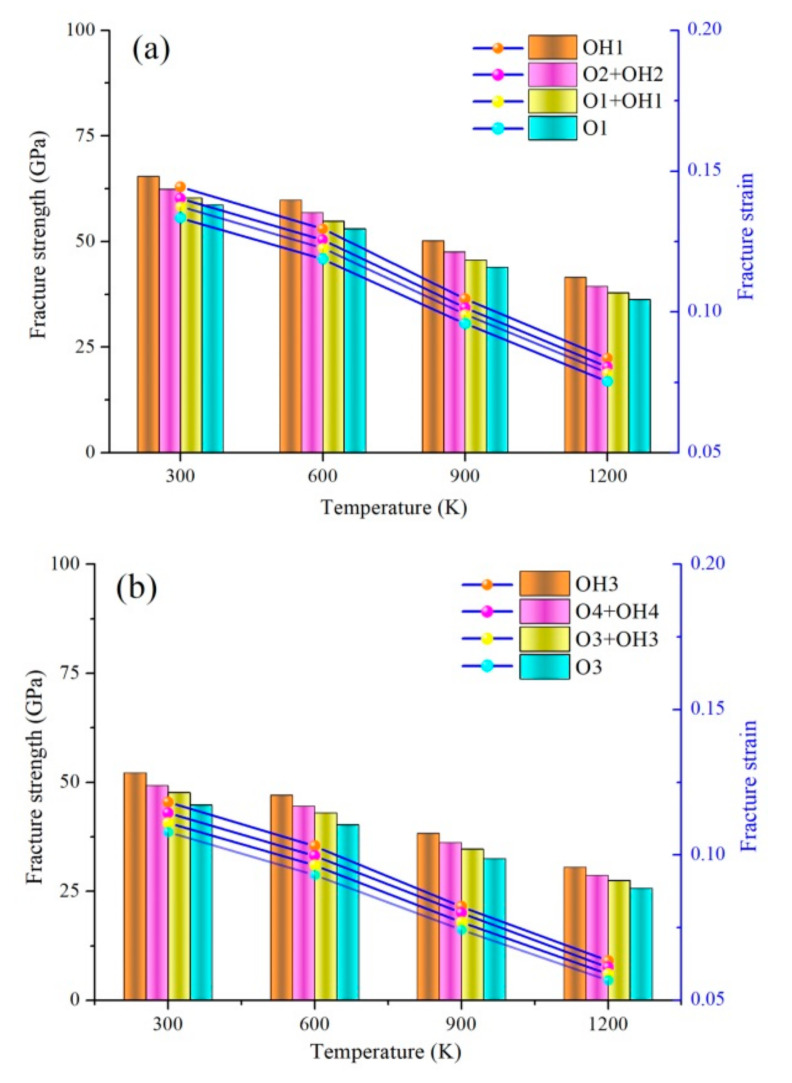
The fracture strength and strain of GrO-BN-GBs configurations with different cases at different temperatures: (**a**) the FGo density is R = 15%, and (**b**) the FGo density is R = 30%.

Under the complex high-temperature environment, the mechanical behaviors of h-BN matrix ceramic, such as strength and constitutive relation, are quite different from those in the normal temperature environment, and show obvious temperature dependence. The h-BN matrix ceramic used as external protection or structure undergoes complex thermal and mechanical coupling. Therefore, temperature dependence of interfacial bonding, mechanical properties, and configuration transition in Gr/h-BN containing grain boundaries and functional groups were investigated. The eight types of FGo density, including R = 15% epoxy (O1), R = 15% hydroxyl (OH1), R = 10% epoxy + R = 5% hydroxyl (O1 + OH1), R = 5% epoxy + R = 10% hydroxyl (O2 + OH2), R = 30% epoxy (O3), R = 30% hydroxyl (OH3), R = 20% epoxy + R = 10% hydroxyl (O3 + OH3), and R = 10% epoxy + R = 20% hydroxyl (O4 + OH4), are created in the right Gr region of GrO-BN-GBs configurations. The fracture strength and strain of GrO-BN-GBs configurations with different cases at different temperatures are shown in Figure 1.

Figure 1 shows that the fracture strength and strain of the GrO-BN-GBs configuration with different cases decreased as the temperature rises from 300 K to 1200 K. In considering the FGo density (R), the fracture strength and strain of the GrO-BN-GBs configuration with the FGo density (R = 15%) were the highest values, compared with the mechanical properties of the GrO-BN-GBs configuration with FGo density (R = 30%). Obviously, the presence of FGo leads to weaker mechanical properties in GrO-BN-GBs configurations. The fracture strength and strain of the OH1 model (R = 15%) were 65.44 GPa and 0.1444, respectively, while the fracture strength and strain of OH1 were reduced to 41.58 GPa and 0.0835, respectively, when the temperature was increased from 300 K to 1200 K. When compared with the R = 15%, the presence of the highest FGo density (R = 30%) resulted in a 41.52% and 46.19% reduction in the fracture strength and strain, respectively. In addition, the weakest values of fracture strength and strain were in the O3 model, compared with the three types of FGo (OH3, O4 + OH4, O3 + OH3). When the temperature was 600 K, the fracture strength and strain of the O3 model were 40.24 GPa and 0.0931, respectively. In addition, the fracture strength and strain of the OH3 model were 47.04 GPa and 0.1033, respectively. The highest fracture strength and strain values were obtained in the OH1 and OH3 models, while the lower fracture strength and strain values were achieved in the O1 and O3 models. The FGo types exerted a remarkable influence on the fracture strength and strain of the GrO-BN-GBs configurations. 

**Figure 2:**
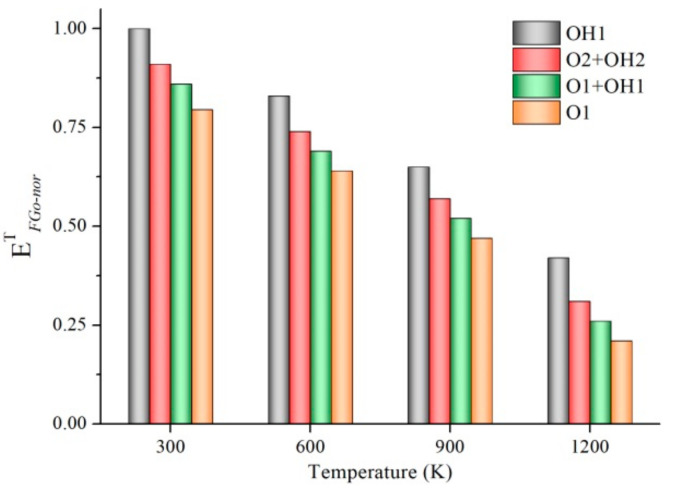
Temperature-dependent energy required for breaking bonds connected with FGo (interfacial bonding energy).

To investigate the disparate impact of FGo types on mechanical values of GrO-BN-GBs configurations at different temperatures, the temperature dependence of interfacial bonding energy is presented in Figure 2.

The temperature dependence of interfacial bonding energy of FGo ($E_{Inter-bonding}$) was computed as shown in the following Equation (1):

(1) $$E_{Inter-bonding}=E_{{}_{GrO-BN-GBs}}^{total}-(E_{BN}+E_{GrO}+E_{GBs})$$

where $E_{GO-BN-GBs}^{total}$ is the total energy of the GrO-BN-GBs configuration, $E_{GrO}$ is the energy of the Gr region, $E_{BN}$ is the energy of the h-BN region, and $E_{GBs}$ is the energy of GBs in the GrO-BN-GBs nanostructure.

The fracture energy of a single FGo ($E_{GO}^{single}$) can be calculated from the total interfacial bonding energy of FGo by Equation (2):

(2) $$E_{GrO}^{single}=\delta E_{Inter-bonding}/N_{GrO}$$

where $N_{GO}$ is the number of FGo in GrO-BN-GBs nanostructure, and δ is the constant of the FGo type.

Finally, the normalized fracture energy ($E_{FGo-nor}$) is calculated by following Equation (3):

(3) $$E_{FGo-nor}=\;\frac{E_{FGo-i}}{E_{FGo-i}^{\max}}$$

where $E_{FGo-i}$ is the i type of FGo in the GrO-BN-GBs nanostructure, and $E_{FGo-i}^{\max}$ is the max value of fracture energy, i=1, 2, 3 or 4.

According to the theory of statistical thermodynamics, the kinetic energy ($E_{K}$) and temperature (T) of all atoms in the system generally meet the Equation (4):

(4) $$E_{K}=\;\sum_{i=1}^{N}\frac{1}{2}m_{i}v_{i}^{2}=\frac{3}{2}\;Nk_{B}T$$

where N is the total atomic number of the GrO-BN-GBs nanostructure, m is the atomic mass, and $k_{B}$ is the Boltzmann constant.

It can be seen from Equation (4) that with the increase in temperature, the total kinetic energy of the system increases, which leads to the thermal vibration of atoms inside the system becoming more intense at high temperatures. Therefore, the freedom probability of the atoms is greater. The temperature-dependent fracture bonding energy ($E_{FGo-nor}^{T}(T)$) can be improved by Equation (5):

(5) $$E_{FGo-nor}^{T}(T)=E_{b}+E_{v}$$

where $E_{b}$ is the elastic deformation energy, and $E_{v}$ is the thermal vibration energy by Equation (6).

(6) $$E_{v}=E_{v}(\Theta ,T)$$

where Θ is the characteristic temperature (such as Einstein temperature or Debye temperature), which essentially reflects the characteristic frequency of the thermal vibration and is a quantity independent of temperature.

Figure 2 shows the temperature-dependent energy required for breaking bonds connected with FGo. The lower values of $E_{FGo-nor}^{T}(T)$ were in the O1 model, compared with OH1, O2 + OH2, and O1 + OH1. In addition, the highest values of $E_{FGo-nor}^{T}(T)$ were in the OH1 model. Clearly, the interfacial bonding ability of the hydroxyl groups is higher than that of the epoxy groups, and the results were consistent with the results of the previous research [34,35]. In addition, $E_{FGo-nor}^{T}(T)$ values decreased with the increase in temperature. As a result, the interfacial bonding ability between the FGo and the matrix was relatively reduced when the temperature was from 300 K to 1200 K. The result was accordant to the temperature dependence of fracture strength and strain, as previously discussed. Moreover, the FGo is more likely to overcome the interfacial binding energy and get out of the inherent equilibrium position, which leads to instability of the GrO-BN-GBs configuration. The more tiny defects that appear in the atomic lattice, the greater the degradation of facture strength and strain. 

### 2.3. Coupling Effect of GBs and FGs at Different Strain 

**Figure 3:**
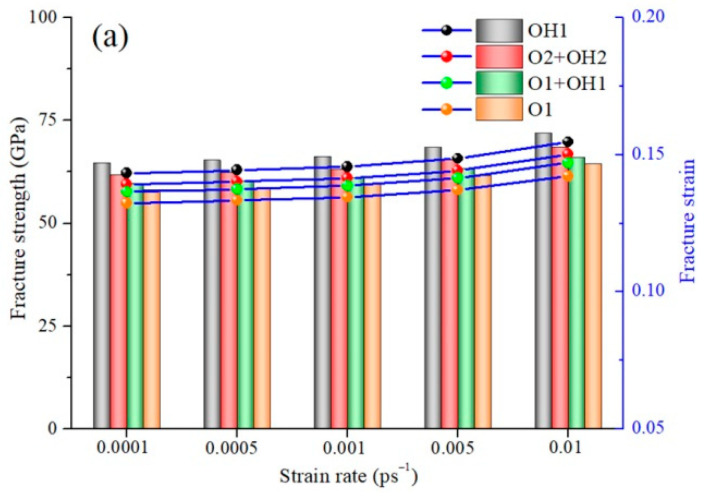

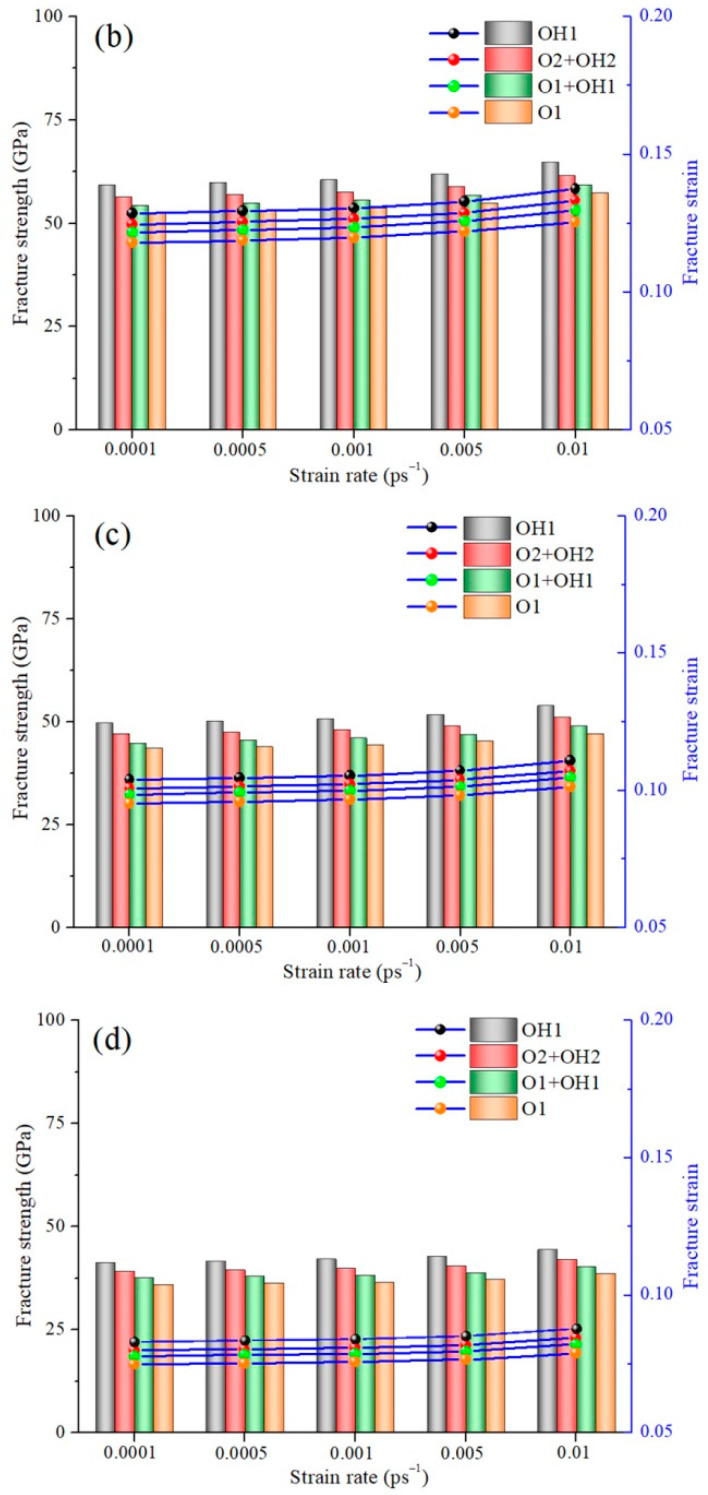
The fracture strength and strain of the GrO-BN-GBs configuration with FGo density R = 15% at different strain rates: (**a**) temperature = 300 K, (**b**) temperature = 600 K, (**c**) temperature = 900 K, and (**d**) temperature = 1200 K.

Ceramic-based materials are also subjected to various dynamic loads or sudden change in strain rate. The influence of sudden change of strain rate on the dynamic mechanical properties of GrO-BN-GBs nanostructures needs to be deeply and systematically studied, as shown in Figure 3. Figure 3 shows the fracture strength and strain of the GrO-BN-GBs configuration with FGo density R = 15% at different strain rates (0.0001, 0.0005, 0.001, 0.005 and 0.01 ps^−1^) and temperatures (from 300 K to 1200 K). The results show the fracture strength and strain increased in the GrO-BN-GBs configuration with the increase in the strain rate. Obviously, the effect of the tensile strain rate on the change of fracture strength and strain was small when the strain rate was in the range of 0.0001 and 0.001 ps^−1^. However, the effect of the strain rate on the mechanical properties of the GrO-BN-GBs configuration suddenly strengthened when the strain rate was larger than 0.001 ps^−1^. The value 0.001 ps^−1^ seems to be a threshold value in the mechanical properties of GrO-BN-GBs configurations. 

An important conclusion is that fracture strength and strain values strongly depend on the interfacial bonding energy of atoms. The dynamic load-induced kinetic energy rapidly increases when the strain rate is larger than the threshold value 0.001 ps^−1^. Then, the kinetic energy of atoms per unit area is greater, especially in the region with weak interfacial bonds. Kinetic energy cannot be uniformly distributed in the whole GrO-BN-GBs configuration, and the chances of uniform dissipation of kinetic energy are reduced, resulting in the energy difference between regions and between atoms. However, when the strain rate is less than the threshold value 0.001 ps^−1^ and the energy difference between regions and between atoms is small, according to the formula (4), the kinetic energy of atoms per unit time is lower. Therefore, it is difficult for atoms to overcome the binding energy, break away from the inherent equilibrium position, and lose stability.

The relationship between fracture strength ($\sigma_{T}$) and the critical energy release rate ($E_{T}$) can be expressed by these Equations (7)–(9):

(7) $$\sigma_{T}=\sqrt{\frac{E_{T}G_{T}}{\pi l}}$$

where l is the crack length, and $G_{T}$ is the temperature-dependent elastic modulus.

(8) $$K_{T}=\sigma_{T}\sqrt{\pi l}$$

where $K_{T}$ is critical fracture toughness.

(9) $$E_{T}=\frac{K_{T}^{2}}{G_{T}}$$

According to Equation (4), the strain rate increases the total kinetic energy of the GrO-BN-GBs nanostructure, and the temperature increases the thermal vibration amplitude of the atoms in the GrO-BN-GBs nanostructure. The strain rate and temperature have a coupling effect on the mechanical properties of the GrO-BN-GBs nanostructure. When the temperature is lower, the temperature effect is not obvious, and the probability of atoms leaving the equilibrium position to form defects is small. The thermal vibration amplitude of atoms increases with the temperature increases. The greater the energy difference generated between regions, the greater the stress concentration.

### 2.4. Interfacial Distance Effect

**Figure 4:**
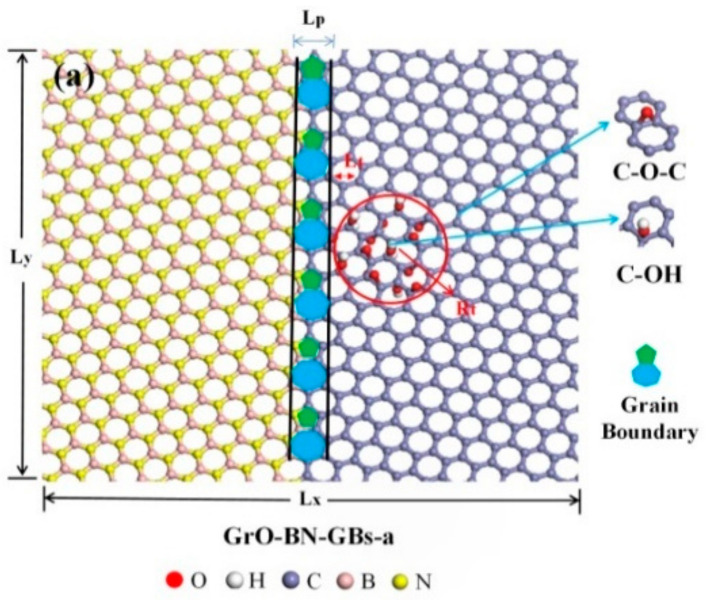

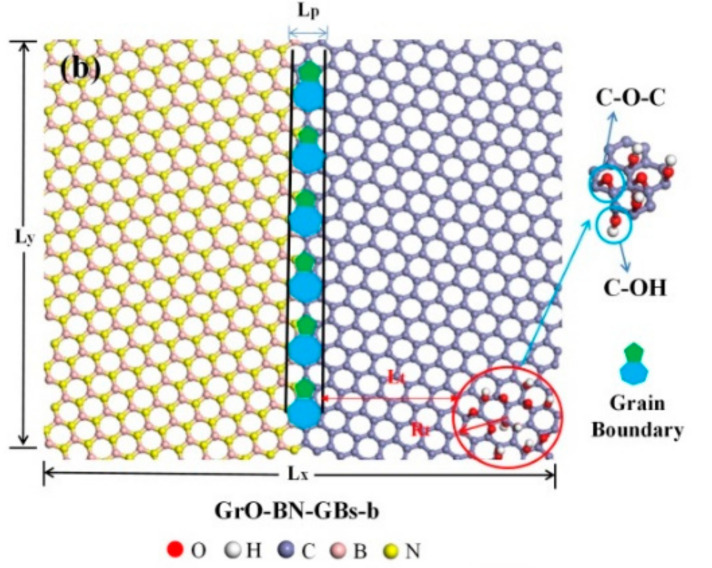
The GrO-BN-GBs configurations with different distances between FGo and GBs: (**a**) the distance between FGo and GBs was less than 3 Å, belonging to the adjacent region (Gro-BN-GBs-a), and (**b**) the distance between FGo and GBs was greater than 20 Å, belonging to the remote region (Gro-BN-GBs-b).

The fracture strain and strength of the GrO-BN-GBs configurations with FGo and GBs followed a general trend with the change of temperature and strain rate. When the distance between GBs (in the interface region) and FGo changes, what is the impact on the interfacial bonding and mechanical properties of the GrO-BN-GBs under multiple physical fields? The GrO-BN-GBs configurations with different distances between FGo and GBs are shown in Figure 4. In this simulated work, the distance between FGo and GBs was divided into two parts: (a) the distance between FGo and GBs was less than 3 Å, belonging to the adjacent region, and (b) the distance between FGo and GBs was greater than 20 Å, belonging to the remote region. The four kinds of FGo were created in each region, including R = 15% epoxy (O1), R = 15% hydroxyl (OH1), R = 10% epoxy + R = 5% hydroxyl (O1 + OH1), and R = 5% epoxy + R = 10% hydroxyl (O2 + OH2).

**Figure 5:**
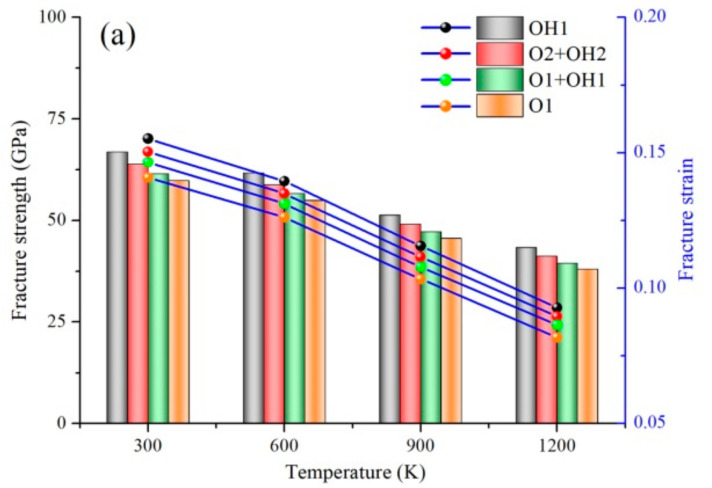

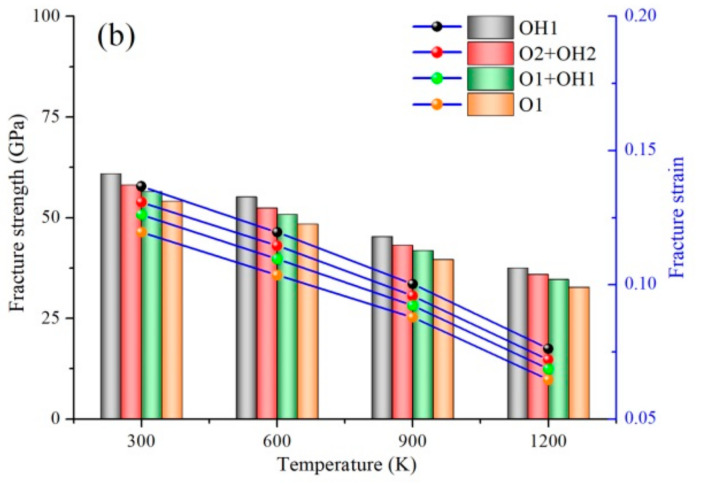
The fracture strength and strain of the GrO-BN-GBs configurations with FGo density R = 15%: (**a**) the GrO-BN-GBs-a model when the distance between FGo and GBs was less than 3 Å and (**b**) the GrO-BN-GBs-b model when the distance between FGo and GBs was greater than 20 Å.

Figure 5 shows the fracture strength and strain of the GrO-BN-GBs configurations with different distances between FGo and GBs at different temperatures (from 300 K to 1200 K). In considering the FGo type in different regions, the highest mechanical values were obtained in the OH1 model, followed by O2 + OH2 model and O1 + OH1. In addition, the worst fracture strength and strain were achieved in the O1 model. In considering the different distances between FGo and GBs, the highest fracture strength and strain of the GrO-BN-GBs configurations were found in the “adjacent region” when the distance between FGo and GBs was less than 3 Å. Nevertheless, the lowest values of the mechanical properties of the GrO-BN-GBs configurations were found in the “remote region” when the distance between FGo and GBs was greater than 20 Å. The mechanical values of the adjacent and remote regions followed a regular trend when the temperature rose from 300 K to 1200 K. The appearance of FGs destroyed the sp^2^ hybridization in the GO-BN-GBs configurations. The original sp^2^ hybridization gradually changed to a hybridization state of sp^2^ + sp^3^. With the shortening of the distance between FGo and GBs, FGo increased the initial strain of atoms near grain boundaries, and then took atoms near grain boundaries out of their equilibrium position. A negative effect on the mechanical properties of the GrO-BN-GBs configurations was produced with the increase in temperature. However, the appearance of the negative effect was delayed when the distance between functional FGo and GBs increased.

### 2.5. Stress Distribution and Configuration Transition

**Figure 6:**
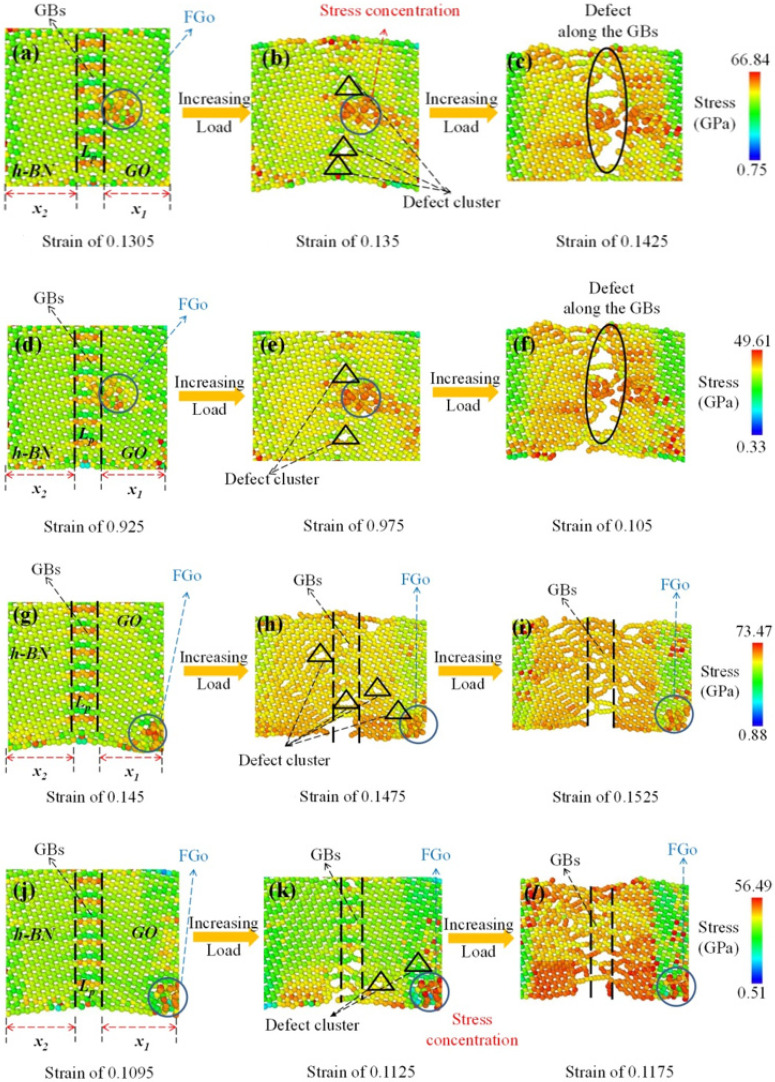
The stress distribution and deformation process of the simulated model, including before cracking, after reaching the strain limit, and after cracking: (**a**–**c**) GrO-BN-GBs-a at a temperature of 300 K, (**d**–**f**) GrO-BN-GBs-a at a temperature of 900 K, (**g**–**i**) GrO-BN-GBs-b at a temperature of 300 K, and (**j**–**l**) GrO-BN-GBs-b at a temperature of 900 K.

In order to understand more clearly the coupling effects of FGo and GBs on the mechanical properties and interfacial bonding of GrO-BN-GBs configurations at different temperatures, the stress distribution and configuration transition of the simulated model, including before cracking, after reaching the strain limit, and after cracking, is shown in Figure 6. The stress distribution of the GrO-BN-GBs configuration was analyzed by von Mises stresses. Von Mises stresses can be described by the Equation (10):

(10) $$\sigma_{Von-mises}=\sqrt{\frac{{\left(\sigma_{1}-\sigma_{2}\right)}^{2}+{\left(\sigma_{2}-\sigma_{3}\right)}^{2}+{\left(\sigma_{1}-\sigma_{3}\right)}^{2}}{2}}$$

where $\sigma_{1}$, $\sigma_{2}$, and $\sigma_{3}$ are defined as the first, secondary, and tertiary principal stresses, respectively.

Figure 6 shows the stress distribution and configuration transition of the simulated model, including before cracking, after reaching the strain limit, and after cracking. Considering the different distances between FGo and GBs, at certain times, the lowest amount of von Mises stresses was obtained in the GrO-BN-GBs configurations when the distance between FGo and GBs was less than 3 Å, while the highest value of von Mises stresses was obtained in the GrO-BN-GBs configurations when the distance between FGo and GBs was greater than 20 Å. With the increasing strain, the von Mises stresses were clearly concentrated around the GBs in the GrO-BN-GBs-a model, and ultimately, failure and defect occurred around the GBs. The increased von Mises stresses at the FGo may be the cause of increased stress at the GBs interface. In addition, the distance between FGo and GBs in the Gro-BN-GBs-a model was greater than that in the GrO-BN-GBs-b model. The von Mises stresses contour appeared in the GBs and FGo region. It was confirmed the FGo increased von Mises stresses in the GrO-BN-GBs-b model, generating a stress concentration phenomenon. Therefore, if the distance between FGo and GBs is too close, there will be a von Mises stresses coupling effect.

The geometric effect also occurred in the GrO-BN-GBs models when the temperature rose. The displacement increment of deflection (d) corresponding to the GrO-BN-GBs model describes the geometric effect.

In this work, the stress and strain in GrO-BN-GBs models were divided into two parts: the Go and h-BN domains. The stress and strain in the Go and h-BN domains of the GrO-BN-GBs models can be calculated by the Equations (11)–(14):

(11) $$\sigma_{Go}=E_{Go\;}(\varepsilon_{Go\;}+\delta_{Go}\sigma_{loading})$$

(12) $$\sigma_{hBN}=E_{hBN\;}(\varepsilon_{hBN}+\delta_{hBN}\sigma_{loading})$$

(13) $$\varepsilon_{Go}=\frac{\sigma_{Go}}{E_{Go\;}}-\delta_{Go}\sigma_{loading}$$

(14) $$\varepsilon_{hBN}=\frac{\sigma_{hBN}}{E_{hBN\;}}-\delta_{hBN}\sigma_{loading}$$

where $\delta_{hBN}$ and $\delta_{Go}$ are the Possion ratios of the h-BN and Gr domains; $\varepsilon_{Go\;}$ is the strain of the Gr domain, and $\varepsilon_{hBN\;}$ is the strain of h-BN domain; $E_{hBN\;}$ and $E_{Go\;}$ are the Young’s modulus of the h-BN and Go domains; $\sigma_{loading}$ is the tension loading.

Geometric effect and deflection (d) caused by the strain of the h-BN and Gr domains can also be calculated by the Equations (15) and (16):

(15) $$d_{1}=\sqrt{{\left(x_{1}+x_{1}\varepsilon_{Go}\right)}^{2}-x_{1}}=x_{1}\sqrt{\varepsilon_{Go}^{2}+2\varepsilon_{Go}}$$

(16) $$d_{2}=\sqrt{{\left(x_{2}+x_{2}\varepsilon_{hBN}\right)}^{2}-x_{2}}=x_{2}\sqrt{\varepsilon_{hBN}^{2}+2\varepsilon_{hBN}}$$

where $d_{1}$ and $d_{2}$ are the displacement increments of deflection in the Gr and h-BN domains, respectively. $x_{1}$ and $x_{2}$ are the lengths of the Gr and h-BN domains, respectively.

**Figure 7:**
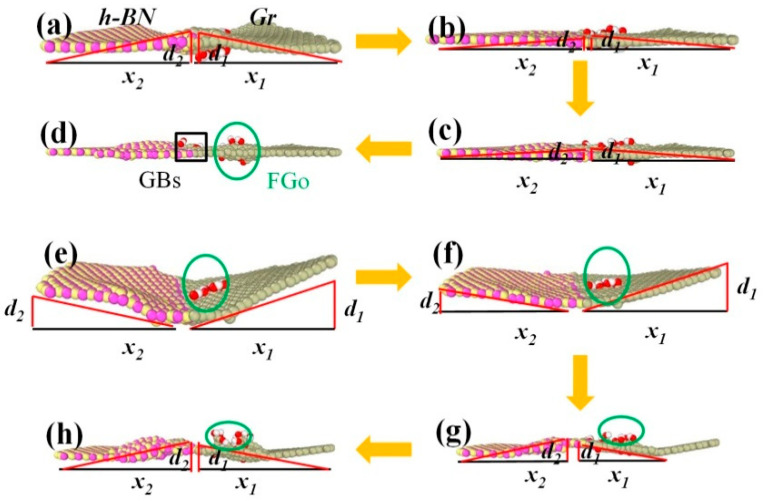
The configuration transition of the simulated model, including before cracking, after reaching the strain limit, and after cracking: (**a**–**d**) GrO-BN-GBs-a at a temperature of 300 K and (**e**–**h**) GrO-BN-GBs-a at a temperature of 900 K. The yellow arrows indicate the increase in strain.

Figure 7 shows the configuration transition of the simulated model, including before cracking, after reaching the strain limit, and after cracking. The deflection increment (d) increased, with the temperature increasing from 300 K to 900 K. The (d) of the GrO-BN-GBs-a model at a temperature of 300 K was lower than that of the GrO-BN-GBs-a model at a temperature of 900 K. A geometric effect was found in the GrO-BN-GBs configuration, which originated from the coupling effect between FGs and grain boundaries. The built-in distortion stress field generated by the coupling effect of temperature and tension loading induce the local geometric buckling of two-dimensional materials. The deflection value (d) perpendicular to the stretching direction decreased with the increase in strain, and the GrO-BN-GBs configuration gradually changed and was “flattened” from the geometric effect (see Figure 7a–d), indicating that the potential energy of the configuration was released during the stretching process. It was also confirmed that temperature and strain would have a weakening effect on the von Mises stresses and deflection value (d) of the GrO-BN-GBs configuration.

## 3. Materials and Methods

In the Y direction, Gr and h-BN are divided into left and right parts. Each part is rotated by certain mismatch angles (θ) and then connected by Equation (17).

(17) $$\theta =\;\theta_{R}+\theta_{L}$$

where $\theta_{R}$ and $\theta_{L}$ are the rotation angles of the left and right parts, respectively.

**Figure 8:**
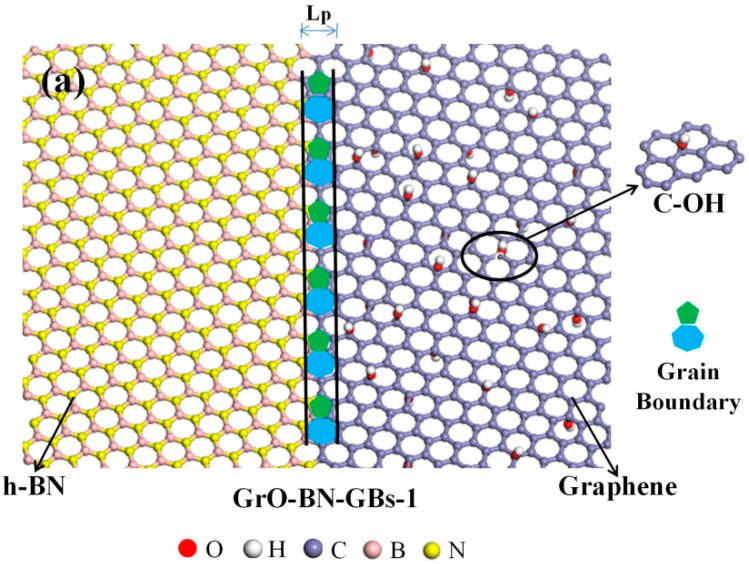

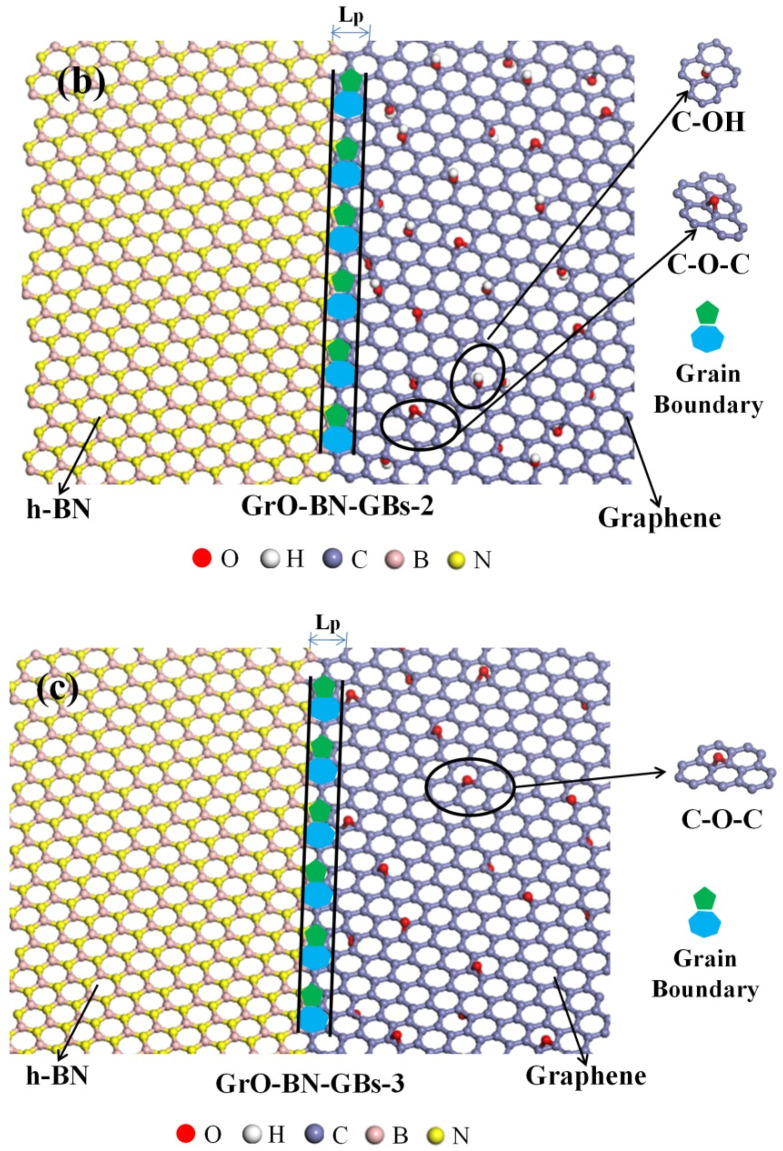
The Gr-BN-GBs configuration in the presence of GBs and FGs: (**a**) plane-view perspective of a portion of a GO-BN-GBs-1 with hydroxyl configuration; (**b**) plane-view perspective of a portion of a GO-BN-GBs-1 with hydroxyl + epoxy configuration; (**c**) plane-view perspective of a portion of a GO-BN-GBs-1 with epoxy configuration.

When $\theta_{R}=\theta_{L}$, symmetric GBs are formed in this simulated model. The periodic length ($L_{p}$) can be described by translation vector pairs on both sides of the GBs. The expression of the mismatch angles and translation vectors on the GBs of G-Hybrid can be described by the following Equations (18)–(20):

(18) $$\theta =\;tan^{-1}[\sqrt{3}m_{L}(m_{L}+2n_{L})]+tan^{-1}[\sqrt{3}m_{R}(m_{R}+2n_{R})]$$

(19) $$L_{R}=\;\alpha_{0}\sqrt{n_{R}^{2}+n_{R}m_{R}+m_{R}^{2}}$$

(20) $$L_{L}=\;\beta_{0}\sqrt{n_{L}^{2}+n_{L}m_{L}+m_{L}^{2}}$$

where $\alpha_{0}$ is the unit vector of the h-BN lattice, and $\;\beta_{0}$ is the unit vector of the Gr lattice. n and m are the translation vector of the system. $n_{R}$ and $m_{R}$ are the translation vector of h-BN, and $n_{L}$ and $m_{L}$ are the translation vector of Gr. I- and IV-type GBs of the Gr-BN configuration are shown in Figure 8.

The graphene/h-BN with FGo and GBs (GrO-BN-GBs) was constructed, shown in Figure 8. The three types of FGo, including epoxy (–O), hydroxyl (–OH) and combination of their type were created at the right Gr region of the GrO-BN-GBs configuration. In addition, the classical configuration of GO, that is, the FGs proportion in GO, is approximately 20–30%, according to experimental reports [30]. The density of FGs in the GO-BN-GBs configuration can be defined as *R* (Equation (21)).

(21) $$R=\frac{N_{\eta }}{N_{c}}$$

where $N_{c}$ is the total number of carbon atoms in the GrO-BN-GBs configuration, and $N_{\eta }$ is the number of carbon atoms connected to the FGo.

The whole work cases were realized by employing a large-scale atomic/molecular large-scale parallel simulator (LAMMPS, Sandia National Laboratories, Germantown, MD, USA) [28].

In this simulation, the atomic trajectory was integrated by the Verlet algorithm. Next, we set the time step to 0.5 fs. The whole work was divided into three aspects: the first step was to run 400 ps in the NPT system at 300 K and 1 atm. The Nosé–Hoover method was chosen to control pressure and temperature. Then at 300 K, it was converted to an NVT ensemble, running for 150 ps; ultimately, whole work ran at the simulated temperature for another 600 ps to generate atomic trajectory information for data analysis. The strain rate of the GO-BN-GBs configuration was 0.0005 ps^−1^.

The reaction force field potential (ReaxFF) was used to capture the bond dynamics and interatomic interactions between hydrogen, oxygen, and carbon atoms, together with the parameters proposed by V. Akarsh et al. [36,37]. The Tersoff potential was applied to describe the short-range interactions of N, B, and C atoms [38]. The interactions of boron and oxide are described by Tersoff parameterization, which was studied by L. Deng [39]. The interactions of nitrogen and oxide are described by charge-optimized many-body (COMB) potential, which was studied by T. Liang et al. [40]. The number of hydrogen-nitrogen and hydrogen-boron bonds were very high, and only the reaction self-consistent force field can be used to accurately simulate this [41].

## 4. Conclusions

Presented herein is the investigation of the mechanical properties, interfacial bonding, and configuration transition of graphene/hexagonal boron nitride coupled by grain boundaries and functional groups under the conditions of high temperatures and strain fields. It was found from the simulation results that the presence of grain boundaries and functional groups can significantly decrease the fracture strength and strain. This is mainly due to configuration transition (from sp^2^ hybridization to sp^2^ + sp^3^ hybridization at the regions linked by functional groups) and the initial strain effect of functional groups. The initial strain effect increases with the decrease in the distance between functional groups and grain boundaries. The lowest amount of von Mises stress was obtained in the GrO-BN-GBs configuration when the distance between the functional groups and grain boundary was less than 3 Å. In contrast, the highest value of von Mises stress was obtained in the GrO-BN-GBs configuration when the distance between the functional groups and grain boundary was larger than 20 Å. In addition, by comparing the different functional group types, the temperature-induced reduction in failure strength, failure strain, and interfacial bonding energy becomes more obvious for the graphene/hexagonal boron nitride coupled by grain boundaries and epoxy groups. A similar observation was obtained for the strain-rate effect, although the strain-rate less than 0.001 ps^−1^ had little effect on the mechanical properties, the strain-rate greater than 0.001 ps^−1^ had a stronger effect on the mechanical properties. Furthermore, the built-in distortion stress field generated by the coupling effect of temperature and tension loading induces the local geometric buckling of two-dimensional materials. Our work provides an understanding of how to control mechanical responses and stability through the grain boundary and functional groups in high temperatures and external loads, which can contribute to new spatial configurations and unprecedented physical properties.

## Figures and Tables

**Table 1:** Comparing the Young’s modulus, failure strength, and strain values of pure graphene obtained by the present study and by previous experimental and computational works.

Various Types	Assessment Method (Potential)	Failure Strain	Failure Strength (GPa)	Young’s Modulus (GPa)	References
Single-layer	MD (Tersoff)	0.204	123	919	Present study
Single-layer	MD (Tersoff)	0.201	131	930	Eshkalak et al. [28]
Single-layer	MD (Tersoff)	0.208	127	925	Fan et al. [33]
	Experimental	0.130	90	1000	Zhao et al. [31]

**Table 2:** Comparing the Young’s modulus, failure strength, and strain values of pure boron nitride obtained by the present study and by previous experimental and computational works.

Various Types	Assessment Method (Potential)	Failure Strain	Failure Strength (GPa)	Young’s Modulus (GPa)	References
Single-layer	MD (Tersoff)	0.207	125	921	Present study
Single-layer	MD (Tersoff)	0.200	131	930	Eshkalak et al. [28]
Single-layer	MD (Tersoff)	0.210	128	923	Fan et al. [33]
	Experimental	-	-	881	Bosak et al. [32]

## Data Availability

Most of the data are presented in the study. The data not presented in this study are available upon request from the corresponding author.

## Abbreviations

Gr	graphene
h-BN	hexagonal boron nitride
G-BN	graphene/boron nitride heterostructures
G-BN-GB	graphene/h-BN with grain boundaries
G-BN-GO	graphene/h-BN with functional groups
FGo	functional groups
GBs	grain boundary
GrO-BN-GBs	graphene/h-BN with FGo and GBs
MD	molecular dynamics
–O	epoxy
–OH	hydroxyl
